# (OLDER) PEOPLE REMEMBER HOW YOU MAKE THEM FEEL: AGE DIFFERENCES IN THE EFFECTS OF SOCIAL EXCHANGES

**DOI:** 10.1093/geroni/igz038.632

**Published:** 2019-11-08

**Authors:** Madeline M Marello, Julie Hicks Patrick, Abigail M Nehrkorn-Bailey

**Affiliations:** West Virginia University, Morgantown, West Virginia, United States

## Abstract

Socioemotional Selectivity Theory poses that as we age our motivations transition from knowledge focused to emotionally focused (Carstensen, 1995). This shift to emotional motivation increases the relevance of relationships and social interactions for older adults. We examined different aspects of social support: frequency of positive/negative social interactions, satisfaction with positive social interactions, and bothered by social interactions -- to investigate these effects on one’s global well-being. Negative and positive social exchanges are linked to psychological health (Newsom et al., 2005), however one’s perceptions of those social interactions are important to consider as well -- being satisfied or bothered by social interactions shows a better perspective of the individual’s experience than simply recording frequency. The results of our multi-group path analysis show that there are different effects of social supports on global well-being contingent on age, consistent with socioemotional selectivity theory. For adults under 30 years old (Mage = 24.0, range 18 to 29) social support did not significantly relate to well-being. For adults over 30 and under 50 (Mage = 38.9) frequency of positive social interactions is significantly related to well-being (B = .201). For adults over 50 (Mage = 58.8, range 50 to 87) the perception of social exchanges, not their frequency, are what influence well-being: both satisfaction with positive social interactions (B = .402) and being bothered by negative social interactions predict well-being (B = -.193). It is important to know that older adult’s perceptions of social exchanges effect their well-being, future directions are discussed.

